# Feedback on the Rate and Depth of Chest Compressions during Cardiopulmonary Resuscitation Using Only Accelerometers

**DOI:** 10.1371/journal.pone.0150139

**Published:** 2016-03-01

**Authors:** Sofía Ruiz de Gauna, Digna M. González-Otero, Jesus Ruiz, James K. Russell

**Affiliations:** 1 Department of Communications Engineering, Faculty of Engineering, University of the Basque Country, Bilbao, Bizkaia, Spain; 2 Department of Emergency Medicine, Oregon Health & Science University, Portland, Oregon, United States of America; Erasmus Medical Centre, NETHERLANDS

## Abstract

**Background:**

Quality of cardiopulmonary resuscitation (CPR) is key to increase survival from cardiac arrest. Providing chest compressions with adequate rate and depth is difficult even for well-trained rescuers. The use of real-time feedback devices is intended to contribute to enhance chest compression quality. These devices are typically based on the double integration of the acceleration to obtain the chest displacement during compressions. The integration process is inherently unstable and leads to important errors unless boundary conditions are applied for each compression cycle. Commercial solutions use additional reference signals to establish these conditions, requiring additional sensors. Our aim was to study the accuracy of three methods based solely on the acceleration signal to provide feedback on the compression rate and depth.

**Materials and Methods:**

We simulated a CPR scenario with several volunteers grouped in couples providing chest compressions on a resuscitation manikin. Different target rates (80, 100, 120, and 140 compressions per minute) and a target depth of at least 50 mm were indicated. The manikin was equipped with a displacement sensor. The accelerometer was placed between the rescuer’s hands and the manikin’s chest. We designed three alternatives to direct integration based on different principles (linear filtering, analysis of velocity, and spectral analysis of acceleration). We evaluated their accuracy by comparing the estimated depth and rate with the values obtained from the reference displacement sensor.

**Results:**

The median (IQR) percent error was 5.9% (2.8–10.3), 6.3% (2.9–11.3), and 2.5% (1.2–4.4) for depth and 1.7% (0.0–2.3), 0.0% (0.0–2.0), and 0.9% (0.4–1.6) for rate, respectively. Depth accuracy depended on the target rate (*p* < 0.001) and on the rescuer couple (*p* < 0.001) within each method.

**Conclusions:**

Accurate feedback on chest compression depth and rate during CPR is possible using exclusively the chest acceleration signal. The algorithm based on spectral analysis showed the best performance. Despite these encouraging results, further research should be conducted to asses the performance of these algorithms with clinical data.

## Introduction

Sudden cardiac arrest is defined as the sudden cessation of the mechanical activity of the heart, confirmed by the absence of signs of circulation. In North America and Europe, death from sudden cardiac arrest has an incidence of about 50 to 100 per 100000 population every year [1], and survival to hospital discharge is poor (less than 10% on average). The International Liaison Committee on Resuscitation (ILCOR) establishes the actions that should be conducted to treat patients in cardiac arrest. These actions are represented by the chain of survival [2], which consists of four links: early recognition of the emergency, early bystander cardiopulmonary resuscitation (CPR), early defibrillation, and early access to advanced care. CPR and defibrillation are the fundamental components of the chain. CPR involves chest compressions that maintain a small critical blood flow to the brain and the myocardium, and increases the likelihood of a successful defibrillation. Survival of ventricular fibrillation cardiac arrest can be doubled or tripled by performing CPR [3, 4].

Current resuscitation guidelines emphasize the importance of providing high quality chest compressions, that is, with a depth of at least 5 cm (but no more than 6 cm) and a rate of between 100 and 120 compressions per minute, allowing chest recoil between compressions and minimizing interruptions [5]. However, studies showed that even trained rescuers often provided too slow and too shallow chest compressions with many interruptions both in hospital [6] and out of hospital [7]. This suggested the need for new strategies to improve CPR quality, such as feedback devices for real-time monitoring and to provide assistance to rescuers, and also for a posteriori debriefing sessions [8]. In the last decade several feedback systems have been developed, and there is evidence of their contribution to improve adherence to recommendations for high-quality CPR during training and in the clinical practice [9].

The first CPR devices were based on pressure/force sensors, assuming a linear relation between the applied force and the achieved compression depth [10]. However, differences in chest stiffness among individuals and during the course of the resuscitation attempt proved this assumption erroneous in humans [11]. More recent devices are based on accelerometers, and calculate the compression depth from the double integration of the acceleration of the chest during CPR. Inbuilt processors integrate the acceleration numerically using algorithms such as the trapezoidal rule. However, integration is a process inherently unstable: the accumulation of integration errors with time results in a significant drift in displacement that impedes accurate estimation of the compression depth [12, 13]. A strategy to solve this problem consists in fixing adequate boundary conditions at the onset/offset of each compression. Some devices include additional force/pressure sensors to identify these points [12, 14]. Increased accuracy could be achieved with a chest compression artefact detector on an additional ECG channel [15]. Other reference signals correlated with the displacement such as the force, the blood pressure, or the transthoracic impedance could also improve depth calculations [16]. However, all of the aforementioned solutions increase the complexity of the feedback device. Another alternative to accelerometers is the use of electro-magnetic signals to measure the chest displacement [17]. In this context, we recently proposed a new algorithm for computing chest compression rate and depth using exclusively the acceleration signal [18]. This algorithm was based on the analysis of the acceleration during chest compressions in the frequency domain, and did not require any additional reference signal for calculating the feedback parameters.

In this study we illustrate the limitations associated with double integration of the acceleration signal to determine compression depth, and describe and compare the performance of three CPR feedback techniques based on the analysis of the acceleration in the time and frequency domains, without using reference signals. Our approach could give rise to simpler, smaller, and less expensive feedback devices.

## The problem of double integration

### Mathematical concepts

By definition, acceleration is the first derivative of velocity with respect to time, and velocity is the first derivative of displacement with respect to time. Conversely, acceleration can be integrated once to obtain velocity, and again to obtain displacement:

$$v\left(t\right)=v\left(0\right)+\int_{0}^{t}a\left(\tau \right)\;d\tau$$ (1)

$$s\left(t\right)=s\left(0\right)+\int_{0}^{t}v\left(\tau \right)\;d\tau$$ (2)

The terms *v*(0) and *s*(0) represent the initial conditions, that is, the velocity and the displacement at the instant *t* = 0, respectively. Every system that performs an integration is intrinsically unstable, as not every bounded input results in a bounded output.

When acceleration is measured and digitized, integration has to be performed numerically. There are several discrete integration algorithms available, the most common ones being rectangular integration, the trapezoidal rule and Simpson’s method. Because of its trade-off between simplicity and accuracy, the trapezoidal rule is the most widespread. This rule can be implemented as a discrete linear filter in which the output is the integrated signal (in this case, the velocity), with the following difference equation:

$$v\left[i\right]=v\left[i-1\right]+\frac{a[i]+a[i-1]}{2}\cdot Ts,$$ (3)

where *Ts* represents the sampling period in seconds, i.e., the inverse of the sampling frequency for the acquisition of the acceleration signal. Likewise, displacement can be computed filtering the velocity:

$$s\left[i\right]=s\left[i-1\right]+\frac{v[i]+v[i-1]}{2}\cdot Ts$$ (4)

Note that even for the first point of the dataset an *i* − 1 term is required, which corresponds to the initial condition for the integration.

**Fig 1:**
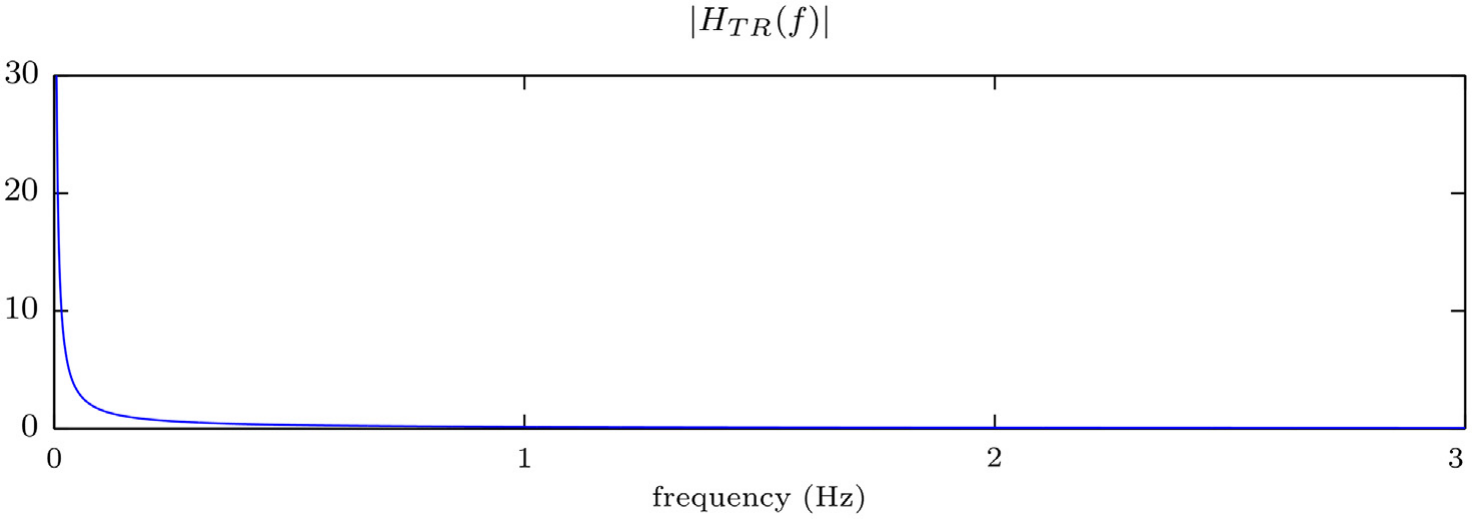
Magnitude of the frequency response of the filter that implements the trapezoidal rule.

Fig 1 shows the magnitude of the frequency response of the linear system that implements the trapezoidal rule. This filter presents a low-pass characteristic that abruptly attenuates the high frequencies of the signal. Conversely, the frequency response |*H*_TR_(*f*)| becomes infinite for *f* = 0 (DC component). This implies that a bounded constant input signal would result in a ramp output that grows to infinity with time. This effect illustrates the instability of the system.

### Drift of the integrated signal

Real-world acceleration recordings usually comprise so called baseline offsets: small errors in the reference level of motion that may be caused by several reasons, including instrumental instability, background noise or calibration errors. Any offset of the acceleration signal (constant or with a slow variation), when doubly integrated, will cause an error that grows quadratically with time. Additionally, inaccuracies in the acceleration signal may result in a drift in the response, particularly in the regions where noise is unbalanced, acting as a small DC offset.

**Fig 2:**
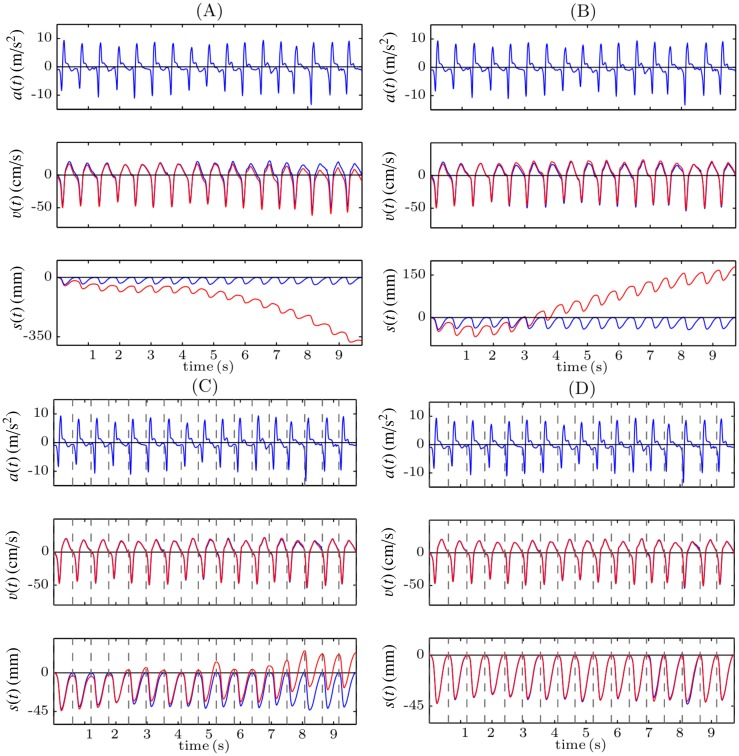
Examples of different strategies to perform the double integration. Direct integration (A), suppression the DC component of the acceleration (B), reset of the initial condition for the velocity after each compression (C), and reset of the initial condition both for the velocity and for the displacement (D).

Fig 2A illustrates the problem with double integration using 10 seconds of a record acquired while CPR was provided to a manikin. The acceleration signal (top panel) and the reference compression depth signal obtained from a displacement sensor (bottom panel, blue line) were registered. The second panel shows the velocity signal computed applying the trapezoidal rule to the acceleration signal (red), and the reference velocity signal, computed differentiating the reference compression depth signal (blue). As can be seen in Fig 2A the integration errors accumulate, and during the last seconds the computed velocity presents a noticeable offset with respect to the reference signal. When the trapezoidal rule is applied again, these inaccuracies lead to big errors in the computed displacement (third panel, red line), of more than 35 cm after only 10 seconds in this case. One strategy to reduce integration errors would be to suppress baseline offsets. When the DC component of the acceleration signal (its mean value) is suppressed before the integration (Fig 2B), the computed velocity signal better replicates the reference one (there is no discernible offset in the last seconds of the record), but there is still a drift in the computed depth of more than 15 cm after 10 seconds. This is because for each compression cycle velocities are not perfectly balanced about the baseline.

In order to reduce the accumulation of the integration errors, the integration could be performed for small signal segments, for example for each compression cycle. For this purpose, it is necessary to identify the onset and offset of each compression and to reset the integration applying appropriate initial conditions after each cycle. Fig 2C shows the result of the integration when the onset and the offset of each compression, depicted by a dashed grey line, is identified in the reference compression depth signal, and when the initial condition for the velocity is set to *v*(*t*) = 0 at these points. In this case, the drift in the computed compression depth signal is significantly reduced, but it still increases with time. In order to solve this problem, the initial condition for the displacement should also be reset to *s*(*t*) = 0 at the beginning of each compression. In this case (Fig 2D) the integration errors are no longer accumulated and there is no drift in the estimated compression depth signal. The limitation of this strategy is that the information related to the chest release, that is, the actual position of the chest after each compression, is lost. This is because the initial conditions are always set to *s*(*t*) = 0 (assuming total release) after each compression, as the actual depth value is unknown. Additionally, a reference signal is required to identify the onset and the offset of each chest compression. This reference signal could be for example the compression force, acquired with a force sensor. However, this would increase the complexity and the cost of the system.

In this context, the aim of this paper was to analyze three alternatives to compute the depth and rate of the chest compressions during CPR using only the acceleration signal.

## Materials and Methods

### Experimental set-up

**Fig 3:**
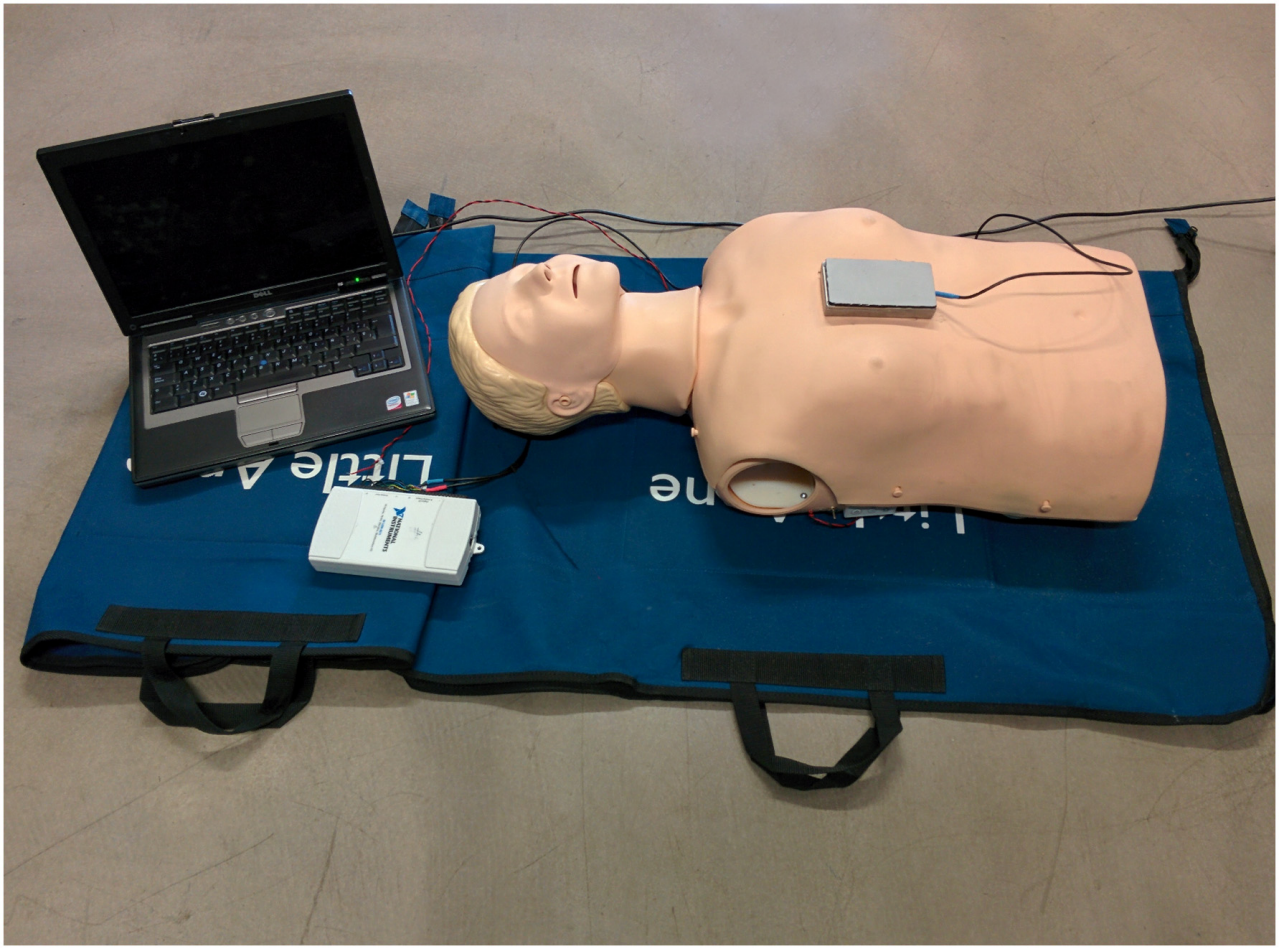
Experimental set-up. Resusci-Anne manikin equipped with a photoelectric sensor (inside the manikin), tri-axial accelerometer encased in a metalic box positioned over the manikin’s chest, acquisition card and laptop computer.

A Resusci Anne manikin (Laerdal Medical, Norway) was equipped with a photoelectric sensor (BOD 6K-RA01-C-02, Balluff, USA) to register the instantaneous compression depth (CD) signal for gold standard computation. Chest compressions were delivered in the center of the manikin’s chest with a tri-axial accelerometer (ADXL330, Analog Devices, USA) placed beneath the rescuer’s hands. The CD signal and the three axes of the acceleration were digitized and recorded using a National Instruments acquisition card (USB NI 6211, USA) connected to a laptop computer, with a sampling rate of 100 Hz and a 16-bit resolution. Fig 3 shows the experimental set-up.

Twenty-eight volunteers received basic chest-compression-only CPR training prior to participating in the recording sessions. They provided their written informed consent, and the ethical committee of the University of the Basque Country (UPV/EHU CEID) approved the experimental protocol. The approval certificate and a translated copy of the informed consent form are available as supporting information.

During the recording sessions volunteers were grouped in couples. Four 10-min episodes were recorded per couple, each one with a different target compression rate (80, 100, 120 and 140 compressions per minute), that was guided by a metronome. The target compression depth was always 50 mm, and was guided using a custom-made computer program based on the reference depth signal recorded by the manikin’s sensor. Volunteers alternated providing 2 min CPR series during each episode, each series involving 30 compressions with 5 s pauses in between. We compiled a total of 56 records. Data were analyzed anonymously.

### Methods

In this section we describe three methods to estimate the chest compression depth and the chest compression rate during CPR applying signal processing techniques exclusively to the acceleration signal.

#### Linear filtering (LF)

As discussed before, the system that applies the trapezoidal rule is unstable. Low frequency components of the input signal will be multiplied by the almost infinite gain that the filter presents around 0 Hz (which is exactly infinite if the input contains a DC component).

**Fig 4:**
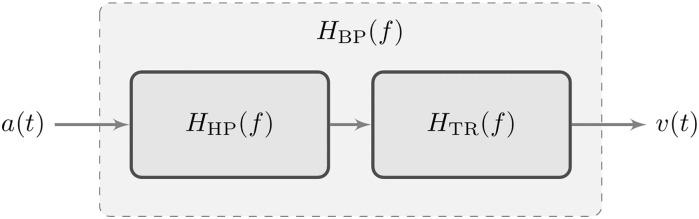
Block diagram of the stable band-pass system.

**Fig 5:**
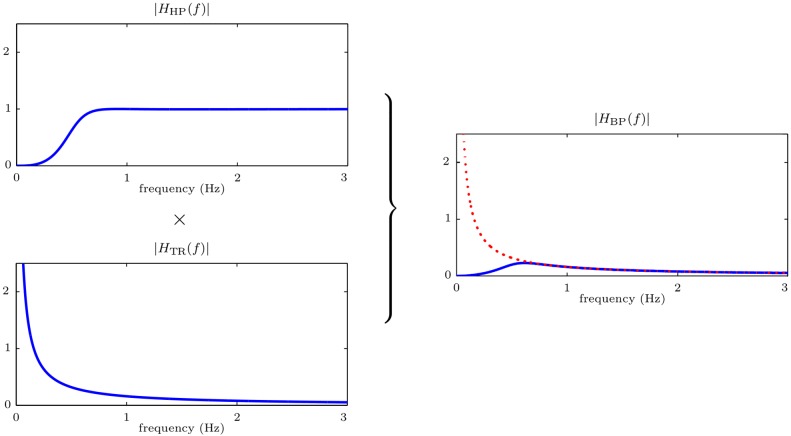
Frequency response of the band-pass filter.

The basis of our first approach is to approximate the integration by a stable band-pass filter. This system is the series connection of a high-pass filter and the trapezoidal rule (low-pass) filter (Fig 4). The purpose of the high-pass filter is to equalize (compensate) the instability for low frequencies, introducing a zero gain at *f* = 0 Hz. The equivalent frequency response of the system, *H*_BP_(*f*), is plotted in Fig 5. It is the result of multiplying the frequency responses of the high-pass filter, *H*_HP_(*f*), and of the trapezoidal rule, *H*_TR_(*f*). Notice that for frequencies above 0.6 Hz the system matches the ideal response (depicted with a red dotted line).

**Fig 6:**
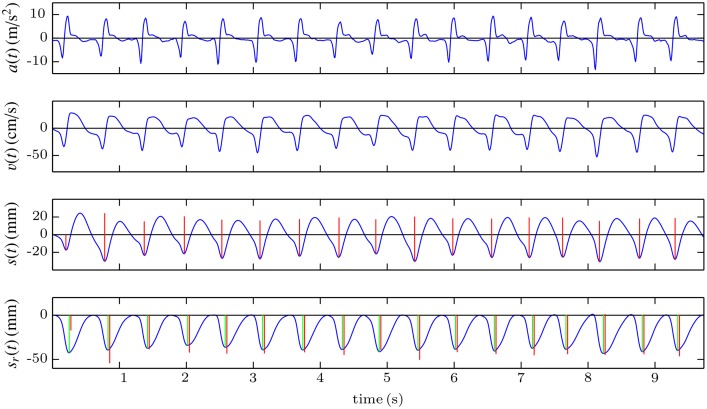
Graphical example of the LF method, based on band-pass filtering.

Fig 6 shows an example of computation of the compression depth signal with this method. The first step consists in processing the acceleration signal (first panel) with the band-pass filter once to obtain the velocity (second panel). This process is then repeated with the velocity to obtain the compression depth signal (third panel). Compared to the reference, this signal has a different waveform, and more importantly, the information relative to a possible leaning of the rescuer (no chest release between compressions) is lost. This is due to the suppression of the low-frequency components of the acceleration after the filtering process. However, the rate and the depth of the chest compressions can be easily computed by applying a peak detector and measuring the peak-to-peak amplitude and the distance between the peaks. The detected compressions and their corresponding depth are depicted by vertical red lines in the third and fourth panels. In the bottom panel, the computed values are compared to the reference values (green lines) obtained from the recorded compression depth signal, *s*_*r*_(*t*). Due to the transient response of the filter, in the first two compressions the errors in the estimation of the compression depth are high.

#### Detection of zero-crossing instants in the velocity signal (ZCV)

**Fig 7:**
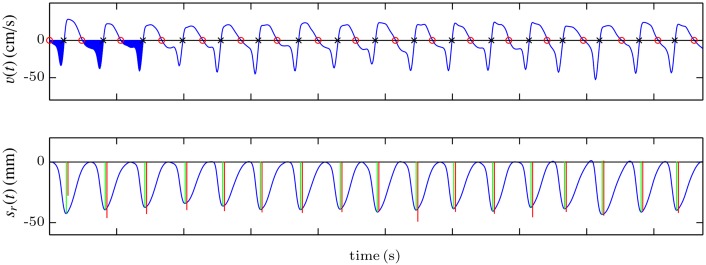
Graphical example of the ZCV method, based on the analysis of velocity.

The basic idea of this method is to calculate the compression depth and rate values directly from the velocity signal, without computing the compression depth signal. First, the band-pass filter described in the previous section is applied to the acceleration to obtain the velocity signal. This signal is quite stable, and can be analyzed to identify the instants corresponding to the onset of each compression cycle and the points of maximum displacement of the chest, as shown in Fig 7. For that purpose, the zero-crossing instants of the velocity signal when it goes from positive to negative (onset of each compression, marked by red circles in the top panel of Fig 7) and when it goes from negative to positive (maximum displacement point, marked by black crosses in the figure) are identified. Then the compression depth corresponding to each cycle is computed as the area of the velocity signal between the onset and the maximum displacement point. In Fig 7 the corresponding area is filled in blue for the first three compressions. In the bottom panel, the computed values (red lines) are compared to the reference values (green lines), and drawn over the reference compression depth signal. The frequency of the chest compressions can be computed as the inverse of the interval in seconds between two zero-crossing instants from positive to negative.

#### Spectral analysis of the acceleration signal (SA)

In order to provide feedback to the rescuer it is not necessary to compute the depth and rate of each compression; an average value every certain period of time could support effective feedback. In this third method neither the compression depth nor the velocity signal are computed by integrating. Rather, the average chest compression depth and rate are computed every 2 seconds by applying spectral analysis to the acceleration signal. The reader is encouraged to refer to a previous study where a more exhaustive and technical analysis of the different constructive parameters of the algorithm was conducted [18].

We provide here a brief description of the basics of the method. During short intervals with continuous chest compressions, the acceleration and the displacement are almost periodic signals whose fundamental frequency is the mean frequency of the compressions. Consequently, they can be approximated by their periodic representation, denoted by *a*(*t*) for the acceleration and *s*(*t*) for the displacement signal in what follows. For an analysis interval of duration *T*_*w*_ seconds, these periodic representations can be modelled using the first *N* harmonics of their Fourier series decomposition (without DC component):

$$a\left(t\right)=\sum_{k=1}^{N}A_{k}\cos\left(2\pi kf_{cc}t+\theta_{k}\right)$$ (5)

$$s\left(t\right)=\sum_{k=1}^{N}S_{k}\cos\left(2\pi kf_{cc}t+\phi_{k}\right)$$ (6)

were *f*_*cc*_ (Hz) is the mean frequency of the compressions, and *A*_*k*_ (m/s^2^), *θ*_*k*_ (rad) and *S*_*k*_ (mm), *ϕ*_*k*_ (rad) are the amplitudes and phases of the *k*-th harmonic of the acceleration and the depth, respectively. Since the acceleration is the second derivative of the displacement, the amplitudes and phases of *a*(*t*) and *s*(*t*) are related by the following equations:

$$S_{k}=\frac{A_{k}}{{\left(2\pi kf_{cc}\right)}^{2}}\cdot 1000\;\text{and}\;\phi_{k}=\theta_{k}+\pi ,\;\text{for}\;k=1,2,\ldots ,N$$ (7)

which can be used to reconstruct *s*(*t*) once *f*_*cc*_, *A*_*k*_ and *θ*_*k*_ are obtained from the acceleration signal. The mean rate expressed in compressions per minute (cpm) and the mean depth (peak-to-peak) of the compressions within the analysis interval are then:

$$\text{rate}\;\text{(cpm)}=f_{cc}\cdot 60$$ (8)

depth(mm)=max{s(t)}-min{s(t)} (9)

**Fig 8:**
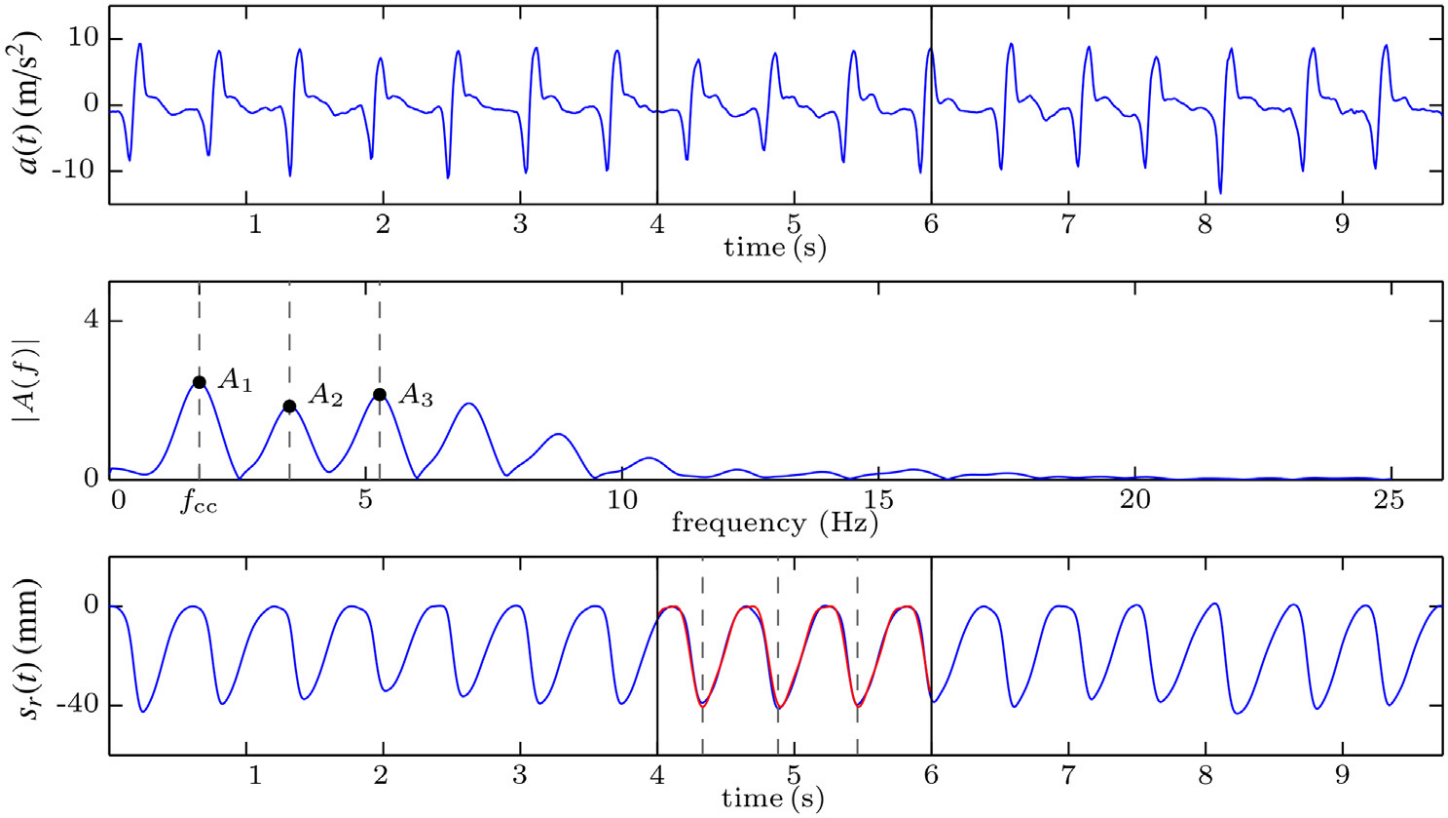
Graphical example of the SA method, based on the spectral analysis of the acceleration.

Based on this mathematical model feedback on the mean rate and depth for each analysis interval of duration *T*_*w*_ were obtained following these steps:

- The acceleration signal was windowed to select the analysis interval and its Fast Fourier Transform (FFT) with zero padding was computed. In the example shown in Fig 8, the selected window is delimited in the first panel with black vertical lines (seconds 4 to 6 of the acceleration).
- The amplitude spectrum of the acceleration was obtained from the FFT. The fundamental frequency, *f*_*cc*_, and the first three harmonics of the acceleration (*N* = 3 in Eq 5) were identified using peak detection. Their amplitudes and phases were obtained from the FFT (middle panel of Fig 8).
- Eq 7 were used to obtain the amplitudes and phases of the first three harmonics of *s*(*t*).
- The displacement for the average compression cycle, i.e., one period of *s*(*t*), was reconstructed applying Eq 6.
- Eqs 8 and 9 were used to obtain the mean rate and depth for the analysed interval.

The third panel of Fig 8 shows the reference compression depth signal (blue) and the reconstructed signal for the selected window (red). This signal is periodic, so it has the same amplitude for all the compressions, that represent the average compression depth during the analysis window.

## Performance evaluation

To evaluate the performance of each method, we calculated the error as the difference between the estimated depth and rate and the gold standard (GS) values. First, we applied an automatic peak detector to the reference compression depth signal. For each identified peak (i.e., for each chest compression), the depth was computed as the peak-to-peak amplitude of the fluctuation (depicted by green lines in the bottom panels of Figs 6 and 7. The rate was computed as the inverse of the distance in seconds between two consecutive compressions, multiplied by 60 to convert the units to cpm. These values were the GS for the LF and the ZCV methods because they provide one value of depth and rate per chest compression. The SA method, however, gives one value of depth and one value of rate every 2 seconds, consequently, the GS for this method was computed by averaging the depth and rate values of all the compressions existing in the corresponding 2-s analysis window.

The distribution of the error in depth was analyzed using boxplots, while histograms and Bland-Altman plots were used to study the error in rate. Additionally, median and percentiles of the unsigned error (absolute and percent values) were measured. The influence of the target rate and the rescuer couple in the depth estimation was also studied.

Friedman’s test analysis of variance was applied to the error sequences to perform between-groups comparisons. Differences in global errors by method, and for each of the methods by rate and by rescuer couple were evaluated (*p*-values < 0.05 were considered statistically significant).

## Results

**Fig 9:**
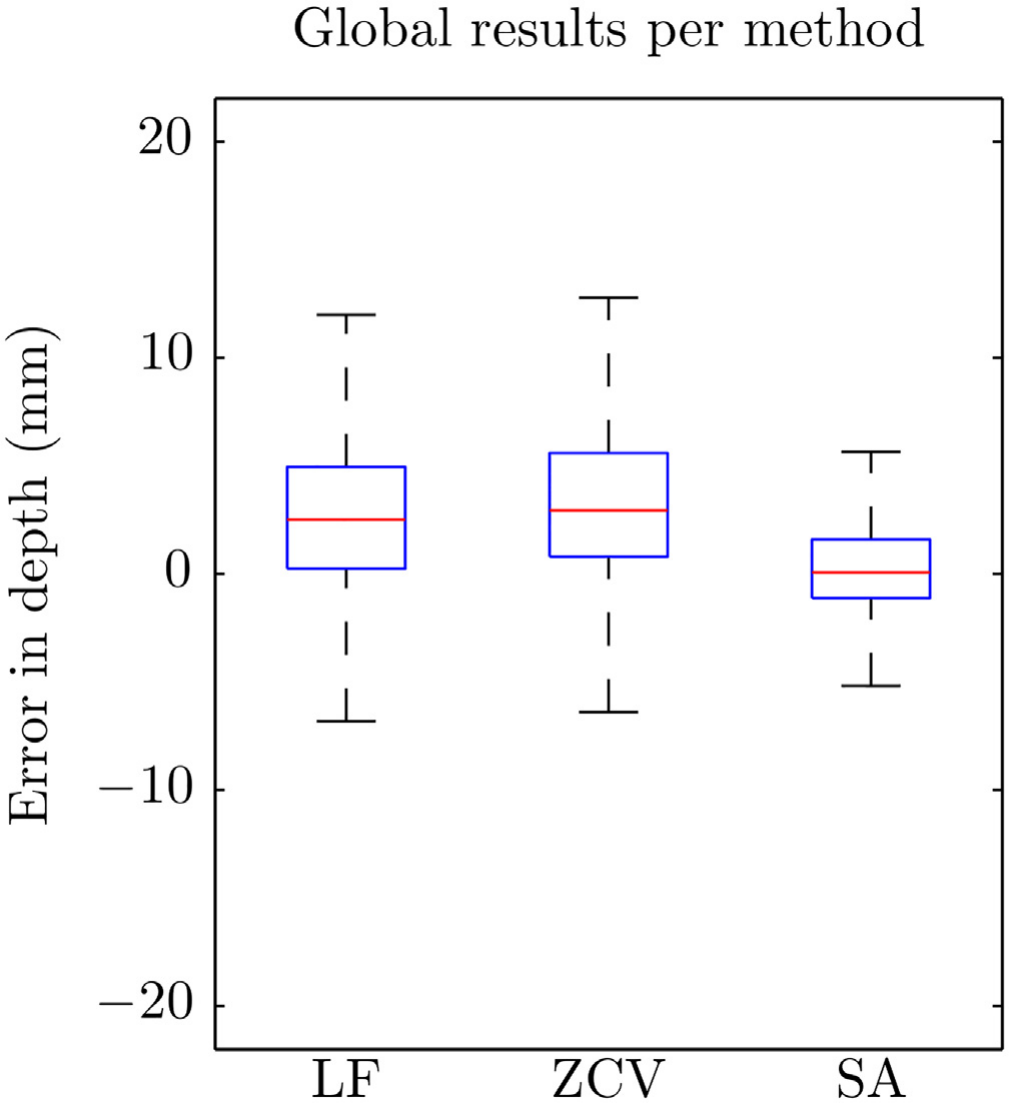
Boxplots of the global errors in depth for the three methods.

Fig 9 shows the boxplots of the errors in the estimation of compression depth for each of the methods. There were statistically significant differences in the errors by method (*p* < 0.001). The LF and the ZCV methods had a slight tendency to overestimate depth values, and the errors were smaller for the SA method. Table 1 shows median and percentile values for the unsigned errors. Median (IQR) unsigned absolute error was 3.1 (1.5–5.4), 3.4 (1.5–6.0) and 1.3 mm (0.6–2.3) for LF, ZCV and SA, respectively. The corresponding percent error was 5.9 (2.8–10.3), 6.3 (2.9–11.3), and 2.5% (1.2–4.4).

**Table 1:** Median values and i-percentiles, Pi, of the unsigned error obtained in the estimation of the compression depth for each method. Values are expressed in mm. LF: linear filtering; ZCV: zero-crossing velocity; SA: spectral analysis.

	Median	P_25_	P_75_	P_90_	P_95_
LF	3.1	1.5	5.4	8.5	11.2
ZCV	3.4	1.5	6.0	9.5	12.9
SA	1.3	0.6	2.3	4.0	5.9

**Fig 10:**
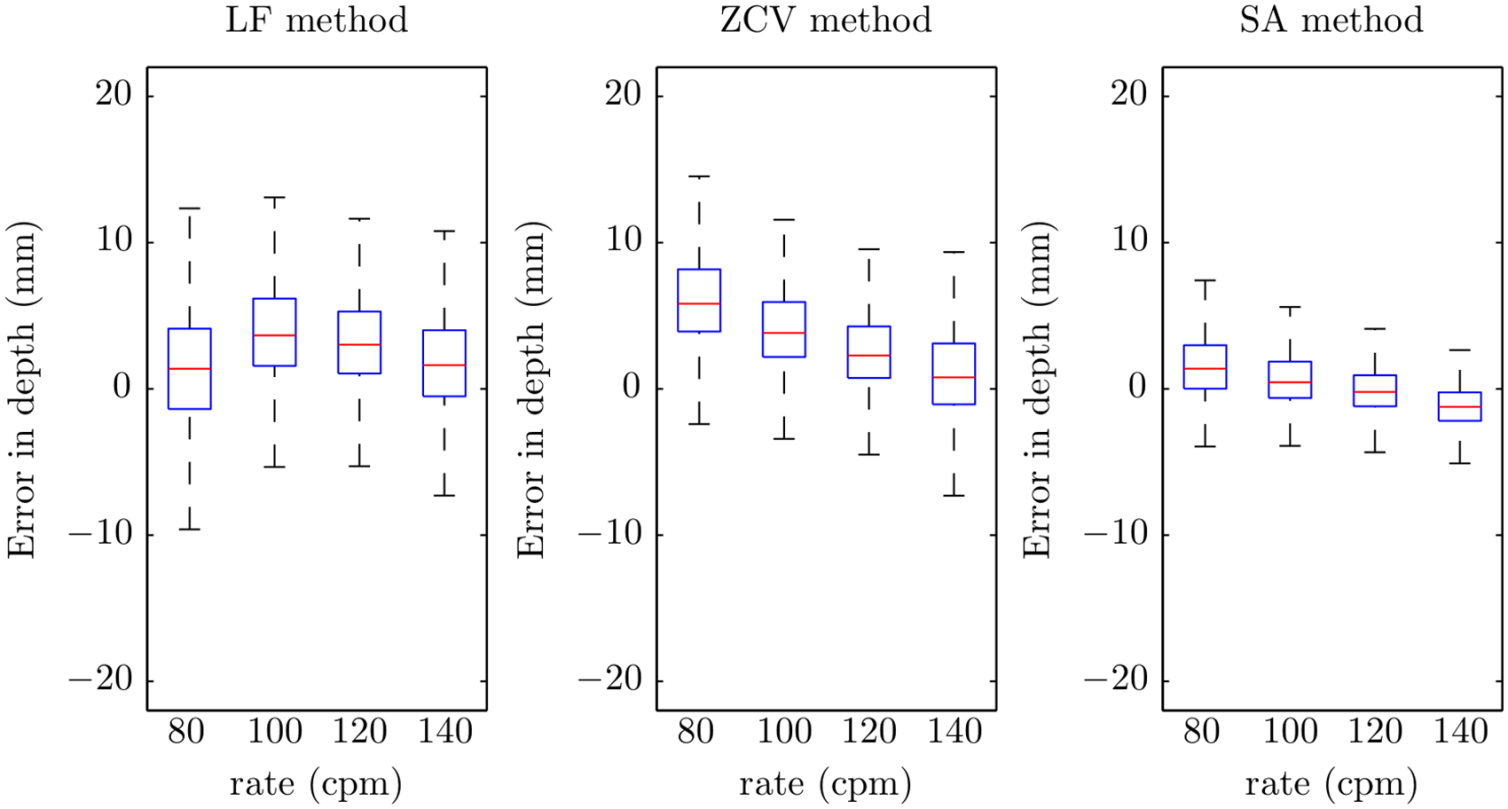
Boxplots of the errors in depth for the three methods by target rate.

Fig 10 shows the boxplots of the errors in depth per target rate. There were significant differences for the three methods (*p* < 0.001). In general, the tendency was to have lower errors for higher chest compression rates. This trend is confirmed by the statistics of the unsigned errors per target rate shown in Table 2.

**Table 2:** Median values and i-percentiles, Pi, of the unsigned error in the estimation of the compression depth with respect to the different target compression rates. LF: linear filtering; ZCV: zero-crossing velocity; SA: spectral analysis.

		Unsigned error (mm)				
	rate (cpm)	Median	P_25_	P_75_	P_90_	P_95_
LF	80	2.9	1.4	4.9	7.4	9.3
	100	3.9	2.0	6.3	9.9	12.8
	120	3.3	1.7	5.5	8.4	11.0
	140	2.6	1.1	4.9	8.0	11.1
ZCV	80	5.8	3.9	8.2	12.1	16.0
	100	3.9	2.3	6.0	9.6	13.1
	120	2.6	1.3	4.5	7.7	10.4
	140	2.1	0.9	4.6	8.7	12.2
SA	80	1.6	0.8	3.0	5.5	7.3
	100	1.1	0.5	2.1	4.0	6.0
	120	1.1	0.5	1.8	2.8	3.8
	140	1.5	0.7	2.4	3.8	5.7

**Fig 11:**
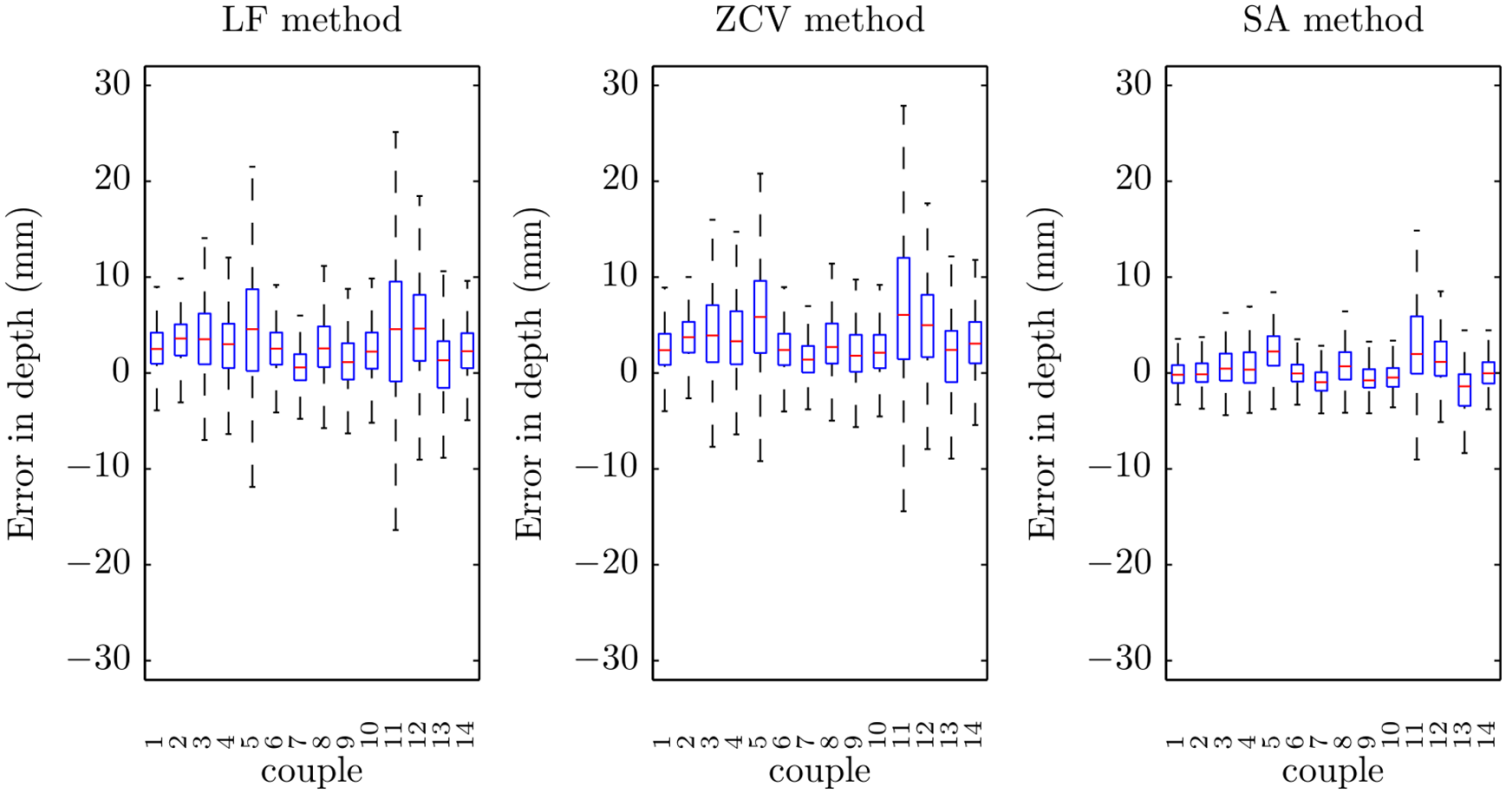
Boxplots of the errors in depth for the three methods by rescuer couple.

Finally, Fig 11 shows the errors of each method in depth estimation by rescuer couple. There were significant differences in the performance of each method for different couples (*p* < 0.001). Some of them presented higher errors in the estimation of the depth for all the methods.

**Fig 12:**
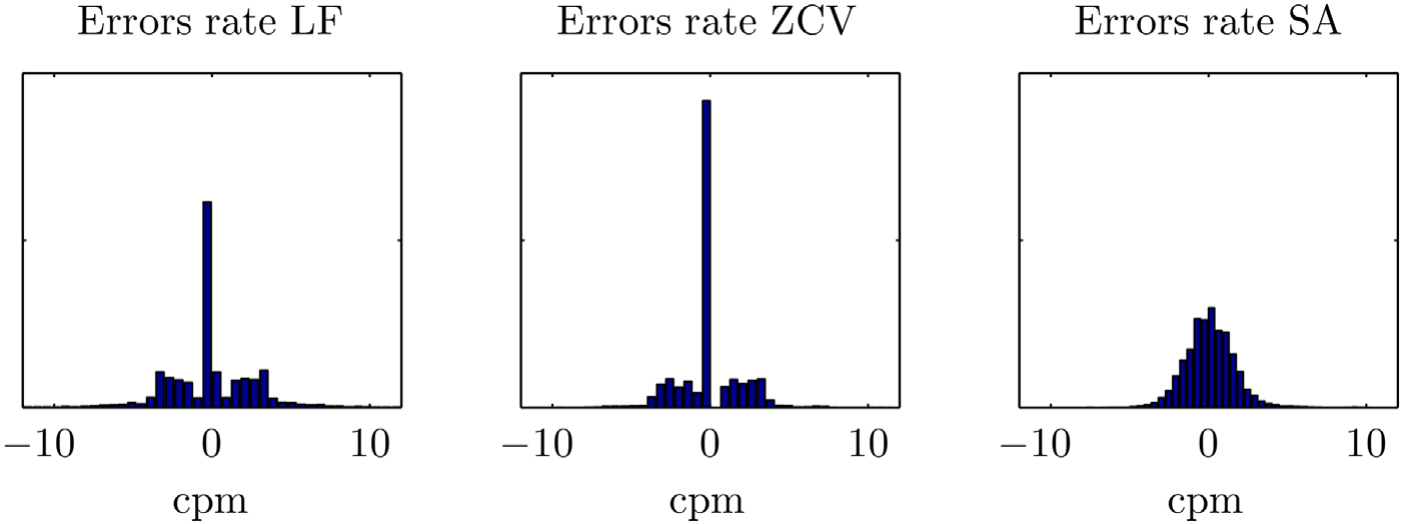
Histograms of the global errors in rate for the three methods.

**Fig 13:**
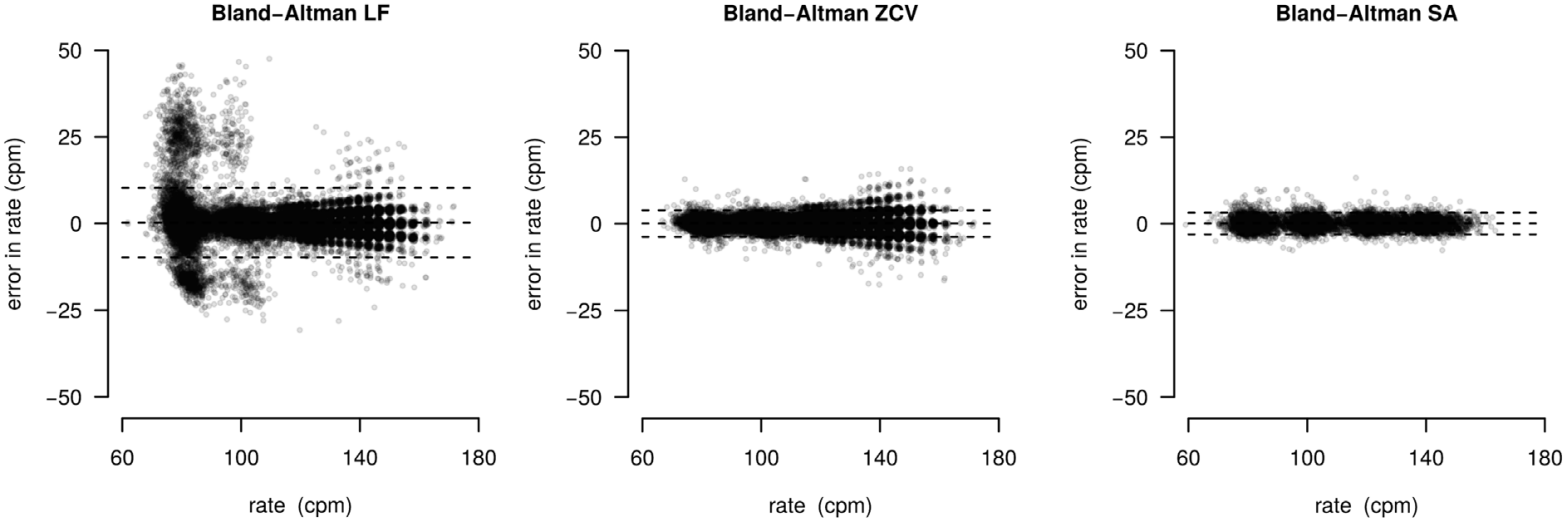
Bland-Altman plot of the errors in rate for the three methods.

Fig 12 shows the histograms of the errors in rate estimation. Median unsigned absolute error was 1.7 cpm (0.0–3.1), 0.0 cpm (0.0–2.3), and 0.9 cpm (0.0–3.1) for LF, ZCV and SA, respectively (*p* < 0.001). The corresponding percent error was 1.7% (0.0–2.3), 0.0% (0.0–2.0), and 0.9% (0.4–1.6). The time-domain methods (LF and ZCV) presented a very pronounced peak in zero, while the errors for the SA method followed a wider normal-like distribution. The LF and ZCV methods presented some high errors, and the SA method had the lowest 75, 90 and 95 percentiles for the unsigned errors (see Table 3). Fig 13 shows Bland-Altman plots for the rate estimation. The LF method presented some very high errors, particularly for low rates. The ZCV and the SA methods presented lower errors, and with a smaller variation by rate.

**Table 3:** Median values and i-percentiles, Pi, of the unsigned error obtained in the estimation of the compression rate for each method. Values are expressed in cpm.

	Median	P_25_	P_75_	P_90_	P_95_
LF	1.7	0.0	3.1	4.8	8.5
ZCV	0.0	0.0	2.3	3.2	3.5
SA	0.9	0.4	1.6	2.4	2.9

## Discussion

This study assesses the accuracy of three strategies for feedback on the depth and rate of the chest compressions by processing exclusively the acceleration signal. To simulate a realistic basic life support scenario volunteers were grouped in couples and series of 30 compressions with alternated 5-s pauses were provided on a manikin, with a reliable reference compression depth signal.

The LF and the ZCV methods reported important errors in the estimation of the chest compression depth. In both approaches the error was above 5 mm in 25% of the compressions, and they tended to overestimate the depth. However, the SA method was very accurate, with an error above 5 mm in only about 5% of the cases, and it was not biased. Percent errors in depth were higher for the time domain methods (5.9%, 6.3% for LF and ZCV, respectively), compared to the 2.5% for SA. The depth accuracy depended on the compression rate; the errors tended to be higher at lower compression rates. However, this change was smaller for the SA method. Within each method, statistically significant differences were also found between rescuer couples.

The Bland-Altman plots describe the performance regarding to compression rate estimation. The high error of LF method at low target rates is remarkable, the obtained limits of agreement (LOA) were the highest (-9.8, 10.3 cpm). The ZCV method quite improved this drawback (LOA: -3.7, 3.9 cpm). However, the SA method again showed the best results with the lowest LOA (-3.0, 3.2 cpm). The performance of the time domain methods (LF and ZCV) was strongly affected by the filter transient, particularly at the beginning of the compression series. This influence was higher for the LF method, in which the filter is applied twice, and significantly decreased the accuracy in the compression rate estimation. On the contrary, the SA method directly analyzes the acceleration without any filtering, and it performed robustly for a wide range of conditions. In any case, the percent error in rate was very low for the three methods (median of 1.7%, 0.0% and 0.9%, respectively).

In recent years, the ability of the transthoracic impedance signal to provide feedback on the rate and depth of chest compressions has been widely studied [19] as an alternative to accelerometers. This signal is available in all current defibrillators through the defibrillation pads. Chest compressions cause fluctuations in the transthoracic impedance waveform. This effect has been studied to detect pauses in chest compressions [20], and to identify individual chest compressions [21, 22]. A recent study has demonstrated the ability of this signal to provide accurate real-time feedback on the chest compression rate [23]. Unfortunately, the analysis of the TI fluctuations does not provide accurate information on depth [24].

Current technology still relies on accelerometers and double integration to estimate depth. Manufacturers have conceived different solutions for the drift problem often protected by patent rights. Two major companies in the market have developed drift compensations techniques based on either additional force or pressure sensors to detect each compression cycle (CPRmeter stand-alone device by Philips/Laerdal), or on advanced filtering techniques requiring reference signals (Real CPR Help technology by Zoll). The TrueCPR device by PhysioControl based on tri-axial field technology is a recent alternative to accelerometers. All these solutions increase the complexity of the device, limiting its widespread use in the practice, especially for bystanders.

The methods discussed in this paper are based solely on accelerometers and could lead to simpler and cheaper devices. However, they present important limitations: they are not capable of detecting inadequate chest release between compressions and are inaccurate when CPR is applied on a patient lying on a bed or on a soft surface [25]. Detection of leaning during chest compressions is the current major drawback of our proposal, and, in general, of any attempt to derive complete feedback from only accelerometers. We intuit that the information of leaning is inbuilt in the acceleration waveform morphology, but more research is needed to prove this hypothesis. Currently the most reliable alternative requires additional hardware systems for adequate feedback on this quality parameter [5, 8].

Currently, the only feedback system accurate on soft surfaces is the TrueCPR by PhysioControl, based on electromagnetic fields [17]. Accelerometer feedback devices overestimate the compression depth, as they measure the sum of the chest displacement (the true depth) and the mattress displacement. This could cause false “push softer” indications to the rescuer and consequently, too shallow chest compressions, which, according to guidelines may diminish the benefit of CPR. This drawback could be overcome using the SA method in two accelerometers, one placed on the chest and the other at the patient’s back [12, 26, 27]. Each accelerometer would compute and transmit one value of depth every 2 s to a control unit, which would calculate the true depth as the difference between the two transmitted values and provide the proper feedback to the rescuer.

The main limitation of our study is that the algorithms were evaluated using a single manikin under ideal laboratory conditions. CPR in the clinical practice is performed under more challenging conditions, in particular with different patients. To what extent varying patient anatomic features or irregular delivering of chest compressions would affect the device performance should be further analyzed. We are currently planning to test the methods with retrospective records of out-of-hospital cardiac arrest episodes in which a commercial accelerometer device was used for feedback. The data stored in the device will serve as the gold standard in this approach.

High-quality CPR is pivotal for improving survival of cardiac arrest. The use of devices to provide real-time feedback on CPR performance has contributed to achieve the target recommendations during training and in clinical practice. Moreover, the information stored in the devices allows proper post-debriefing which is key for improving interventions. Unfortunately, there is no current evidence on their influence in improving outcome. Consequently, current recommendations state that the use of these devices could help to optimize CPR performance but as a part of an overall strategy to improve CPR quality.

## Conclusion

Accurate feedback on chest compression depth and rate during CPR is possible using only the chest acceleration signal. Among the discussed alternatives, the algorithm based on the spectral analysis of the acceleration provided a very high accuracy and robustness. Devices based only on accelerometers might be simpler and less expensive than commercial existing devices. Nevertheless, despite the encouraging results in a simulated scenario, further research should be conducted to asses the performance of these algorithms with clinical data.

## Supporting Information

S1 File Results corresponding to LF method. Estimated vs. Gold Standard depth and rate values for the whole dataset.
(XLS)

S2 File Results corresponding to ZCV method. Estimated vs. Gold Standard depth and rate values for the whole dataset.
(XLS)

S3 File Results corresponding to SA method. Estimated vs. Gold Standard depth and rate values for the whole dataset.
(XLS)

S4 File Informed consent (translated version). (PDF)

S5 File Ethical committee certificate. (PDF)
