# Supplementary material for: Feedback on the Rate and Depth of Chest Compressions during Cardiopulmonary Resuscitation Using Only Accelerometers
Source: PLoS One. 2016 Mar 1;11(3):e0150139. doi: 10.1371/journal.pone.0150139 (PMC4773040; doi:10.1371/journal.pone.0150139)
Supplement: S4 File — (PDF) [file pone.0150139.s004.pdf]

This is a translated version of the original document (in Spanish)

MEASUREMENT OF CHEST COMPRESSION DEPTH AND ACCELERATION SIGNALS DURING CARDIOPULMONARY RESUSCITATION IN A MANIKIN SCENARIO

Purpose of the research study

Cardiopulmonary resuscitation (CPR) is a key intervention to treat cardiac arrest victims. It consists in applying chest compressions and ventilations to the patient to maintain a minimal flow of oxygenated blood to the vital organs. Quality of chest compressions is critical to maximize survival from cardiac arrest. Feedback systems can help rescuers to improve chest compression quality. The acceleration of the chest during chest compressions can be processed to calculate chest compression rate and depth and provide feedback to the rescuers. The GSC research group is working on the development of new methods to provide feedback on the rate and depth of chest compressions during CPR.

The aim of this study is to record chest compression depth and acceleration signals while chest compressions are being provided to a resuscitation manikin. These records will be used to design and evaluate methods to calculate chest compression depth and rate from the acceleration signal, and to study the characteristics of the displacement and acceleration of the chest during CPR. We need volunteers to provide chest compressions to the manikin in order to introduce variability in the acceleration and compression depth waveforms.

Procedures

1. You will participate in a training session of approximately 90 min. During this session, a member of our team will describe the CPR technique, and you will practice the delivery of chest compressions on a resuscitation manikin. You will also receive a detailed explanation of the procedure that will be followed during the recording sessions.

2. You will participate in one or two recording sessions of approximately 90 min each. During each session several registers will be acquired. For each register you will provide chest compressions to the manikin with a certain target compression rate and depth. You will follow the procedure learned during the training session. A member of our team will guide you and indicate the target compression rate and depth for each register. All information acquired will be kept anonymous and confidential.

Possible risks

The risks involved in the study are minimal. However, you may experience certain discomfort or muscular soreness. To minimize this effect, breaks will be programmed between records. You may also feel frustrated if you fail to achieve the target depth or rate for the chest compressions. During the training session, we will teach you proper CPR technique to help you achieve the target. In any case, the records will be useful for the study even if you fail to achieve target depths and rates.

Benefits and financial considerations

You will not receive economical compensation for your participation in the study. However, you will have the opportunity to learn how to provide CPR, and to improve your technique using a resuscitation manikin. Additionally, you will contribute to the development of methods to provide feedback during chest compressions, which could ultimately derive in an increase of survival to cardiac arrest.

Use of the results

The findings from this study may be published in a scientific conference or journal. You will not be identified as a participant in any case. After the study is completed, you may request information about the study results.

Voluntary participation

Participation in this study is entirely voluntary. You may choose to participate or not. If at any point you do not wish to continue, you have the right to withdraw from the study without negative consequences.

I, ………………………………………………………… declare that I have understood the objectives and purposes of this study. All of my questions and concerns about this study have been addressed. I choose, voluntarily, to participate in this research project. I am aware that I can withdraw at any time without any consequence. I certify that I am at least 18 years of age.

____

print name of participant

____________________________________ ____________________

signature of participant date
