# Supplementary material for: Feedback on the Rate and Depth of Chest Compressions during Cardiopulmonary Resuscitation Using Only Accelerometers
Source: PLoS One. 2016 Mar 1;11(3):e0150139. doi: 10.1371/journal.pone.0150139 (PMC4773040; doi:10.1371/journal.pone.0150139)
Supplement: S5 File — (PDF) [file pone.0150139.s005.pdf]

Universidad del País Vasco / Euskal Herriko Unibertsitatea
NAZIOARTEKO BIKAINTASUN CAMPUSA
CAMPUS DE EXCELENCIA INTERNACIONAL

IKERKETA SAILEKO ERREKTOREORDETZA
VICERRECTORADO DE INVESTIGACIÓN

Mabel Marijuán Angulo, Directora de Ética en la Investigación y la Docencia de la Universidad del País Vasco/Euskal Herriko Unibertsitatea,

CERTIFICA

Que el artículo titulado: *Feedback on the Rate and Depth of Chest Compressions during Cardiopulmonary Resuscitation Using Only Accelerometers,* contiene datos de voluntarios cuya participación fue solicitada a través de documentos de Consentimiento Informado adecuados y que se conservan en un fichero de Protección de Datos cuyo nombre es **INB - RCP MANIQUI**, y que se encuentra dado de alta en la AVPD, cumpliendo así con los requisitos establecidos por la Ley 14/2007 de Investigación Biomédica y por el RD1720/2007 de desarrollo de la Ley Orgánica 15/1999 de Protección de Datos de Carácter Personal.

Para que conste y a petición de la Dra. Sofía Ruiz de Gauna, lo firmo en Leioa a catorce de septiembre de dos mil quince.

IIEB CEID
eman ta zabal zazu
UNIVERSIDAD DEL PAÍS VASCO EUSKAL HERRIKO UNIBERTSITATEA

Mabel Marijuán Angulo
Directora de Ética en la Investigación y la Docencia de la UPV/EHU

BIZKAIKO CAMPUSA
CAMPUS DE BIZKAIA
Sarriena Auzoa, z/g
48940 LEIOA

susi.marcos@ehu.es
www.ehu.es/CEID
